# Evaluation of model performance to predict survival after transjugular intrahepatic portosystemic shunt placement

**DOI:** 10.1371/journal.pone.0217442

**Published:** 2019-05-23

**Authors:** Andrew S. Allegretti, Nathan E. Frenk, Darrick K. Li, Harish Seethapathy, Xavier F. Vela Parada, Joshua Long, Paul Endres, Daniel S. Pratt, Raymond T. Chung, Suvranu Ganguli, Zubin Irani, Kei Yamada

**Affiliations:** 1 Division of Nephrology, Department of Medicine, Massachusetts General Hospital, Boston, MA, United States of America; 2 Division of Interventional Radiology, Department of Radiology, Massachusetts General Hospital, Boston, MA, United States of America; 3 Liver Center and Gastrointestinal Division, Department of Medicine, Massachusetts General Hospital, Boston, MA, United States of America; 4 Department of Medicine, Mount Sinai West and St. Luke’s Hospital, New York, NY, United States of America; Medical University of Graz, AUSTRIA

## Abstract

**Background/Aims:**

The MELD score was developed to predict survival after transjugular intrahepatic portosystemic shunt (TIPS) placement. Given changes in practice patterns and development of new prognostic tools in cirrhosis, we aimed to evaluate common models to predict mortality after TIPS placement.

**Methods:**

Analysis of consecutive patients who underwent TIPS placement for ascites or bleeding. Performance to predict 90-day mortality was assessed by C statistic for six models (MELD, MELD-Na, CLIF-C ACLF, Child-Pugh, Platelet-Albumin-Bilirubin, and Emory score). Added predictive value to MELD score was assessed for univariate predictors of 90-day mortality. Stratified analysis by TIPS indication, emergent placement status, and TIPS stent type was performed.

**Results:**

413 patients were analyzed (248 with variceal bleeding, 165 with refractory ascites). 90-day mortality was 27% (113/413). Mean MELD score was 15 ± 7.9. MELD score best predicted mortality for all patients (c = 0.779), for variceal bleeding (c = 0.844), and for emergent TIPS (c = 0.817). CLIF-C ACLF score best predicted mortality for refractory ascites (c = 0.707). Addition of sodium to the MELD score did not improve predictive value across multiple strata. Addition of hemoglobin improved MELD score’s predictive value in variceal bleeding. Addition of age improved MELD score’s predictive value in refractory ascites.

**Conclusions:**

MELD score best predicted 90-day mortality. Addition of sodium to the MELD score did not improve its performance, though mortality prediction was improved using Age-MELD for ascites and Hemoglobin-MELD for bleeding. An individualized risk stratification approach may be best when considering candidates for TIPS placement.

## Introduction

Transjugular intrahepatic portosystemic shunt (TIPS) placement has been an established therapy for complications of cirrhosis and portal hypertension dating back to 1989, when the procedure was first described for successful treatment of recurrent bleeding varices.[1] While randomized trials have demonstrated survival advantages for TIPS in patients with refractory ascites and variceal bleeding,[2–4] proper patient selection remains vital due to significant risks of post-procedure hepatic encephalopathy, liver failure, and the high overall morbidity/mortality in this population.[5]

To this end, prognostic scores have been developed and refined for multiple cohorts of patients with cirrhosis, including candidates for TIPS. The most widely used tool, the MELD score,[6] was originally developed to predict early death among those undergoing elective TIPS placement.[7] In more recent years, the MELD score has been adapted to include serum sodium and is used to model overall survival for transplant allocation in cirrhosis.[8] Over time, there have been updates to clinical guidelines and adoption of new procedural techniques that have impacted practice patterns and outcomes for TIPS recipients. Furthermore, there are key differences in patient characteristics depending on TIPS indication (e.g., refractory ascites vs. variceal bleeding) and acuity (e.g., emergent vs. elective TIPS referral) that may warrant real-time adjustments to prognostic models based on the relevant clinical scenario. Therefore, we aimed to evaluate six new and/or existing prognostic tools in cirrhosis (MELD score,[6] MELD-Na score,[8] Chronic Liver Failure Consortium Acute on Chronic Liver Failure [CLIF-C ACLF] score,[9] Child-Pugh [CP] score,[10] Platelet-Albumin-Bilirubin [PALBI] score,[11] and Emory score.[12]), stratified by these key clinical factors, in order to find the optimal models to predict 90-day survival after TIPS placement.

## Methods

### Patient population and data collection

We performed a retrospective cohort study of all patients who had a first-time TIPS successfully placed for refractory ascites or variceal bleeding between 1995 and 2016 within a network of acute care hospitals (Partners Healthcare, Massachusetts, USA), including one liver transplant center (Massachusetts General Hospital, Boston, MA) where the majority of TIPS cases were referred. Data were identified using a centralized clinical data warehouse designed for research and quality improvement purposes.[13, 14] TIPS recipients were identified using the ICD-9 code “39.1 –intra-abdominal venous shunt” (or related ICD-10 codes) as well as review of TIPS procedure censuses. At least two authors manually identified all TIPS recipients to confirm the ICD-9/10 code corresponded to a new TIPS placement and the indication was consistent with guideline definitions.[15] Patients were excluded if they had a TIPS placed for other reasons (such as portal vein thrombosis or non-cirrhotic portal hypertension).

### TIPS procedure and clinical care

TIPS placement was performed according to standard clinical practice, in interventional radiology suites under general anesthesia.[16–18] Bare metal stents (WALLSTENT, Boston Scientific, Natick, Massachusetts) were used starting in 1995, and were phased out by 2011. Covered stents (VIATORR, Gore, Flagstaff, Arizona) were used from 2003 to 2016. Patients were admitted for at least 24 hours observation following the procedure, and subsequent outpatient care was provided in interventional radiology and hepatology clinics. All data obtained for this study was taken in the context of routine care and was available in the longitudinal electronic medical record. Laboratory data were taken from the most recent values immediately prior to TIPS, up to 7 days prior to the procedure.

### Prognostic scores and outcomes

Patients were assessed for the primary outcome of death by 90 days after TIPS placement. Stratified analysis was performed by (1) indication for TIPS (variceal bleeding vs. refractory ascites), (2) emergent vs. elective TIPS placement for variceal bleeding, and (3) use of covered vs. uncovered stent. Emergent TIPS was defined as requiring vasopressor support for hemorrhagic shock or use of an esophageal balloon tamponade. Post-TIPS hepatic encephalopathy noted if severe enough to require an unscheduled outpatient or emergency room visit. Patients whose vital status was unconfirmed at 90 days were excluded as being lost to follow-up (n = 6). Patients with hepatic hydrothorax (n = 20) were included in the refractory ascites group, and sensitivity analyses were performed by excluding these patients, as well as comparing them to those with ascites alone.

Six prognostic scores were assessed for their ability to predict 90-day mortality: MELD score, MELD-Na score, CLIF-C ACLF score, CP score, PALBI score, and Emory score. See **S1 Table** for individual score components. Two additional sensitivity analyses were performed for the performance of these six prognostic scores: evaluating one year mortality, and evaluating 90-day transplant-free survival. Individual variables were assessed to predict 90-day mortality in univariate analysis after stratification by TIPS indication. Variables represented in the MELD/MELD-Na score (total bilirubin, INR, serum creatinine, serum sodium) and the top univariate predictors of 90-day mortality were comparatively assessed for discrimination to predict 90-day mortality across multiple patient strata (bleeding vs. ascites, emergent vs. elective, covered vs. uncovered stent) using the methods described below.

### Statistical analysis

For each prognostic score, area under the receiver operating characteristic (AUROC) curves/C statistics were calculated to evaluate model performance. These were compared within each stratum using Chi square testing and were displayed with 95% Wald confidence limits. The incremental improvement of adding individual variables to the MELD score was presented using three independent metrics: (a) direct change in C statistic (receiver operating characteristic contrast estimation using Chi square testing), (b) category-free net reclassification index (NRI) and (c) integrated discrimination index (IDI).[19] All model assumptions were checked and given a normal distribution, parametric testing was used for all univariate and multivariable analysis. Continuous variables were presented as means ± standard deviation. Descriptive tables were stratified by indication for TIPS and analyzed using Student’s t-tests and Chi square tests (or Fisher’s exact test when cells contained less than 5 values) for continuous and categorical variables, respectively. SAS version 9.4 (Cary, NC) was used for all analyses. Two-tailed p values ≤ 0.05 were considered statistically significant.

### Ethics statement

The Partners Institutional Review Board approved this study. The need for informed consent was waived. All procedures and practices abide by the guidelines set forth by the Declarations of Helsinki and Istanbul.

## Results

### Demographics and TIPS characteristics

**Fig 1:**
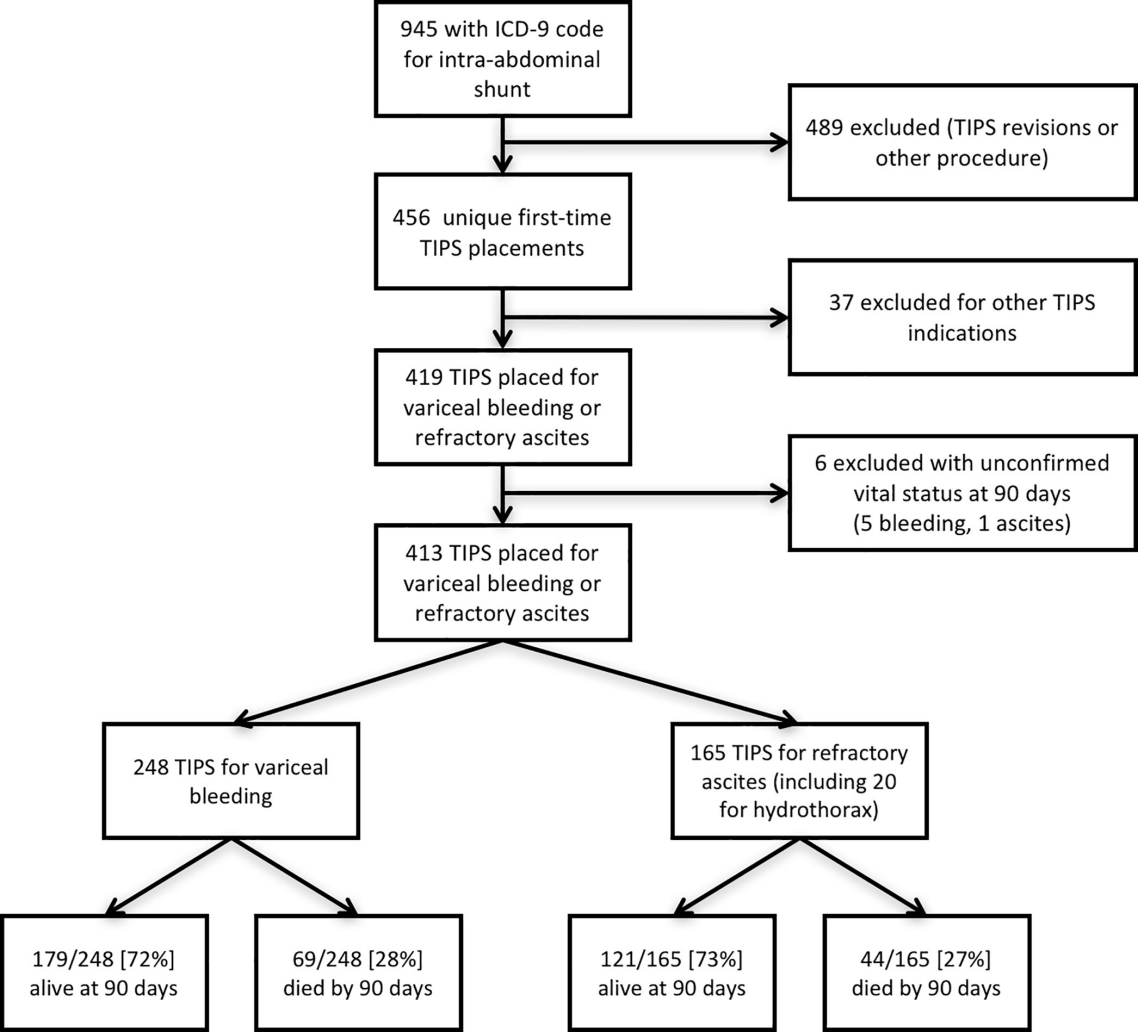
Flow chart of patient selection.

456 patients underwent TIPS creation during the study period. 43 patients were excluded, including 37 first-time TIPS placed for other indications and 6 patients with unverifiable vital status at 90 days. 413 patients were included in the final analysis (see **Fig 1**). 248 TIPS were placed for variceal bleeding and 165 TIPS were placed for refractory ascites. Mean age was 56 ± 11.4 years, 123/413 (30%) were female, and the most common etiology of cirrhosis was alcoholic liver disease (see **Table 1**).

**Table 1:** Demographics and clinical characteristics, by indication for TIPS.

	All Patients (n = 413)	Variceal Bleeding (n = 248)	Refractory Ascites (n = 165)	P value
**Age (years)**	56 ± 11.4	56 ± 12.0	58 ± 10.3	0.05
**Female sex (%)**	124 (30%)	67 (27%)	57 (35%)	0.10
**White race (%)**	342 (83%)	203 (82%)	139 (84%)	0.53
**Non-Hispanic ethnicity (%)**	396 (96%)	240 (97%)	156 (95%)	0.26
**Co-morbidities (%)**				
Diabetes mellitus ^a^	118 (29%)	62 (26%)	56 (35%)	0.05
Chronic kidney disease ^b^	84 (21%)	41 (17%)	43 (27%)	0.02
Cardiovascular disease ^c^	59 (15%)	33 (14%)	26 (16%)	0.51
Hypertension ^c^	133 (33%)	80 (33%)	53 (33%)	0.99
**Etiology of cirrhosis (%) ^m^**				0.19
Non-alcoholic steatohepatits	38 (9%)	19 (8%)	19 (12%)	
Alcohol	135 (33%)	85 (35%)	50 (30%)	
Hepatitis C	72 (18%)	37 (15%)	35 (21%)	
Multifactorial	71 (18%)	48 (20%)	23 (14%)	
Other	88 (22%)	51 (21%)	37 (23%)	
**Prior complications of liver disease (%)**				
Pre-TIPS encephalopathy ^d^	134 (34%)	72 (31%)	62 (40%)	0.05
Gastrointestinal bleeding ^e^	286 (72%)	224 (95%)	62 (39%)	<0.001
Spontaneous bacterial peritonitis ^f^	47 (12%)	13 (6%)	34 (22%)	<0.001
**Laboratory values**				
Sodium (mEq/L)	137 ± 5.7	139 ± 4.8	134 ± 5.5	<0.001
Creatinine (mg/dL)	1.4 ± 0.84	1.2 ± 0.74	1.6 ± 0.95	<0.001
White blood count (K/uL)	7.8 ± 4.86	8.5 ± 5.36	6.8 ± 3.77	<0.001
Hemoglobin (g/dL)	10.1 ± 1.61	10.1 ± 1.61	10.1 ± 1.61	0.95
Platelets (K/uL)	103 ± 63.0	94 ± 50.5	117 ± 76.3	0.001
Albumin (g/dL)	2.7 ± 0.57	2.6 ± 0.55	2.8 ± 0.57	<0.001
International normalized ratio (INR)	1.5 ± 0.39	1.5 ± 0.38	1.5 ± 0.40	0.51
Total bilirubin (mg/dL)	4.0 ± 5.97	4.7 ± 6.72	3.0 ± 4.44	0.002
Aspartate aminotransferase (U/L)	172 ± 688.2	220 ± 876.4	97 ± 154.1	0.03
Alanine aminotransferase (U/L)	76 ± 219.8	89 ± 273.6	56 ± 90.1	0.08
Alkaline phosphatase (U/L)	126 ± 99.7	113 ± 94.7	146 ± 104.1	0.001
**TIPS procedural characteristics**				
Emergent placement (%)^§^	—	61 (25%)	—	—
Pre-TIPS portosystemic gradient (mm Hg) ^g^	18 ± 7.5	18 ± 8.7	17 ± 5.2	0.10
Post-TIPS portosystemic gradient (mm Hg) ^h^	7 ± 3.5	7 ± 3.9	7 ± 3.0	0.99
Reduction in portosystemic gradient (mm Hg) ^i^	11 ± 6.5	12 ± 7.4	11 ± 4.7	0.06
Covered stent (%) ^j^	254 (62%)	146 (59%)	108 (66%)	0.14
Diameter of final TIPS dilation (mm) ^k^	9.9 ± 1.08	9.8 ± 1.11	9.9 ± 1.04	0.67
TIPS revision within 90 days (%) ^l^	49 (12%)	29 (12%)	20 (12%)	0.94
Hepatic Encephalopathy within 90 days (%)^¶^	150 (39%)	91 (38%)	59 (42%)	0.52

Continuous variables are displayed as mean ± standard deviation. P value represents comparison between variceal bleeding and refractory ascites groups. Key: TIPS (transjugular intrahepatic portosystemic shunt)

^a^ n = 403

^b^ n = 400

^c^ n = 401

^d^ n = 389

^e^ n = 396

^f^ n = 391

^g^ n = 384

^h^ n = 402

^i^ n = 382

^j^ n = 410

^k^ n = 407

^l^ n = 399

^m^ n = 404

^**§**^ Emergent placement was only considered among patients receiving TIPS for variceal bleeding.

^**¶**^ Requiring unscheduled outpatient visit or admission to the emergency room/inpatient setting. n = 380

Compared to patients with variceal bleeding, patients with ascites were older (58 ± 10.3 vs. 56 ± 12.0 years; p = 0.05), more frequently had pre-TIPS encephalopathy (40% vs. 31%; p = 0.05) and spontaneous bacterial peritonitis (22% vs. 6%; p < 0.001). Patients with ascites had lower sodium, higher creatinine, lower white blood count, higher platelet count, higher albumin, and lower total bilirubin (see **Table 1**).

Hemodynamic and technical details of the TIPS procedure are described in **Table 1**. For all patients, the mean reduction in portosystemic gradient was 11 ± 6.5 mm Hg. Collateral veins were embolized during 57/411 (14%) of all procedures. A 10-mm diameter stent was used in 353/407 (87%) of cases. Covered stents were used in 254/410 (62%) of cases. Among those with TIPS placed for variceal bleeding, 61/248 (25%) were placed emergently. There were no major differences in procedural characteristics between patients with variceal bleeding and refractory ascites.

### Prognostic scores for 90-day mortality

Overall, 113/413 (27%) patients died by 90 days (69/248 [28%] for variceal bleeding and 44/165 [27%] for refractory ascites; p = 0.38; see **S1 Fig**). 61/248 (25%) of patients with bleeding had TIPS placed emergently. Of these, 28/61 (41%) died by 90 days.

**Table 2** describes mean prognostic scores for all patients, and compares them by TIPS indication. Four of the six scores were available for all 413 patients (n = 389 for CP and Emory scores due to missing pre-TIPS encephalopathy status). For all patients, mean MELD score was 15 ± 7.9, mean MELD-Na score was 18 ± 7.6, mean CLIF-C ACLF score was 40 ± 7.3, mean CP score was 9.3 ± 2.09, mean PALBI score was -2.4 ± 0.72, and mean Emory score was 1.2 ± 1.20. MELD score (p = 0.41) and CLIF-C ACLF score (p = 0.85) were similar between bleeding and ascites groups. Patients with ascites had higher MELD-Na, CP, and PALBI scores (p < 0.001 for all), and had lower Emory scores (p < 0.001).

**Table 2:** Mean scores/values of prediction models, by indication for TIPS.

	All Patients (n = 413)	Variceal Bleeding (n = 248)	Refractory Ascites (n = 165)	P value
**MELD score**	15 ± 7.9	15 ± 8.2	16 ± 7.5	0.41
**MELD-Na score**	18 ± 7.6	17 ± 7.9	20 ± 6.8	<0.001
**CLIF-C ACLF score**	40 ± 7.3	40 ± 7.8	40 ± 6.6	0.85
**Child-Pugh score**^§^	9.3 ± 2.09	8.7 ± 2.03	10.2 ± 1.84	<0.001
**Platelet-Albumin-Bilirubin score**	-2.4 ± 0.72	-2.3 ± 0.72	-2.6 ± 0.71	<0.001
**Emory Score**^§^	1.2 ± 1.20	1.5 ± 1.36	0.8 ± 0.79	<0.001

Variables are displayed as mean ± standard deviation. P value represents comparison between variceal bleeding and refractory ascites groups.

Key: TIPS (transjugular intrahepatic portosystemic shunt), MELD (Model for End Stage Liver Disease), CLIF-C ACLF (Chronic Liver Failure Consortium Organ Failure Acute on Chronic Liver Failure Score

^**§**^ n = 389

### Univariate predictors of 90-day mortality

Due to major baseline differences between patients with variceal bleeding and refractory ascites, analysis of univariate predictors of 90-day mortality were stratified by indication for TIPS placement (see **Table 3**). All 6 prognostic scores significantly predicted 90-day mortality in both bleeding and ascites subgroups (p ≤ 0.01 for all).

**Table 3:** Predictors of vital status at 90 days, stratified by indication for TIPS.

		Variceal Bleeding			Refractory Ascites	
	Alive at 90 days (n = 179)	Died by 90 days (n = 69)	P value	Alive at 90 days (n = 121)	Died by 90 days (n = 44)	P value
**Age (years)**	55 ± 12.2	56 ± 11.7	0.50	57 ± 9.9	61 ± 10.5	0.01
**Female sex (%)**	53 (30%)	14 (20%)	0.14	41 (33%)	15 (36%)	0.71
**White race (%)**	152 (85%)	51 (74%)	0.06	106 (86%)	33 (80%)	0.45
**Non-Hispanic ethnicity (%)**	175 (98%)	65 (95%)	0.16	117 (94%)	39 (95%)	0.85
**Diabetes mellitus (%)**	46 (26%)	16 (24%)	0.76	42 (34%)	14 (37%)	0.76
**Chronic kidney disease (%)**	23 (13%)	18 (28%)	0.008	32 (26%)	11 (29%)	0.72
**Etiology of cirrhosis (%)**	—	—	0.17	—	—	0.84
**Prior complications of liver disease (%)**						
Pre-TIPS encephalopathy	49 (29%)	23 (35%)	0.38	45 (38%)	18 (49%)	0.27
Gastrointestinal bleeding	163 (95%)	61 (92%)	0.38	46 (39%)	17 (42%)	0.67
Spontaneous bacterial peritonitis	6 (4%)	7 (11%)	0.05	27 (23%)	7 (18%)	0.56
**Laboratory values**						
Sodium (mEq/L)	139 ± 4.4	140 ± 5.5	0.08	134 ± 5.0	134 ± 6.7	0.83
Creatinine (mg/dL)	1.0 ± 0.52	1.7 ± 1.0	<0.001	1.4 ± 0.89	1.9 ± 1.01	0.006
White blood count (K/uL)	8.0 ± 5.03	9.6 ± 6.03	0.06	6.5 ± 3.21	7.7 ± 4.96	0.15
Hemoglobin (g/dL)	10.3 ± 1.51	9.6 ± 1.76	0.002	10.2 ± 1.72	9.9 ± 1.26	0.18
Platelets (K/uL)	97 ± 48.5	87 ± 55.0	0.14	122 ± 75.7	103 ± 76.9	0.16
Albumin (g/dL)	2.6 ± 0.56	2.5 ± 0.55	0.05	2.9 ± 0.55	2.8 ± 0.62	0.24
International normalized ratio (INR)	1.4 ± 0.35	1.7 ± 0.39	<0.001	1.5 ± 0.34	1.6 ± 0.51	0.09
Total bilirubin (mg/dL)	3.1 ± 4.10	9.0 ± 9.73	<0.001	2.1 ± 2.05	5.4 ± 7.47	0.007
Aspartate aminotransferase (U/L)	123 ± 231.3	474 ± 1599.9	0.07	91 ± 151.0	115 ± 162.9	0.38
Alanine aminotransferase (U/L)	64 ± 129.9	153 ± 470.3	0.13	50 ± 82.5	71 ± 107.7	0.23
Alkaline phosphatase (U/L)	111 ± 82.6	117 ± 121.1	0.72	146 ± 110.3	145 ± 85.8	0.95
**TIPS procedural characteristics**						
Emergent placement (%)	33 (18%)	28 (41%)	<0.001	—	—	—
Pre-TIPS portosystemic gradient (mm Hg)	18 ± 9.1	20 ± 7.4	0.09	17 ± 4.9	18 ± 5.8	0.58
Post-TIPS portosystemic gradient (mm Hg)	7 ± 3.9	7 ± 3.8	0.81	7 ± 2.9	7 ± 3.3	0.76
Reduction in portosystemic gradient (mm Hg)	11 ± 7.6	13 ± 6.7	0.06	10 ± 4.8	11 ± 4.5	0.32
Covered stent (%)	111 (62%)	35 (51%)	0.10	85 (71%)	23 (53%)	0.04
Diameter of final TIPS dilation (mm)	9.8 ± 1.19	10.0 ± 0.87	0.22	9.9 ± 1.15	9.9 ± 0.68	0.75
**Liver Disease Prediction Models**						
MELD score	12 ± 6.5	22 ± 8.1	<0.001	14 ± 5.9	20 ± 9.5	<0.001
MELD-Na score	14 ± 6.4	23 ± 7.8	<0.001	18 ± 5.7	24 ± 7.9	<0.001
CLIF-C ACLF score	39 ± 7.2	44 ± 8.1	<0.001	39 ± 5.5	44 ± 7.8	<0.001
Child-Pugh score	8.3 ± 1.92	9.8 ± 1.93	<0.001	9.9 ± 1.86	10.8 ± 1.67	0.01
Platelet-Albumin-Bilirubin score	-2.5 ± 0.65	-1.8 ± 0.69	<0.001	-2.7 ± 0.65	-2.3 ± 0.77	0.001
Emory Score	1.2 ± 1.24	2.3 ± 1.40	<0.001	0.7 ± 0.76	1.1 ± 0.83	0.01

Continuous variables are displayed as mean ± standard deviation. See Table 1 for variables with missing data.

Key: TIPS (transjugular intrahepatic portosystemic shunt), MELD (Model for End Stage Liver Disease), CLIF-C ACLF (Chronic Liver Failure Consortium Organ Failure Acute on Chronic Liver Failure Score

Among patients with variceal bleeding, those who died had higher creatinine (1.7 ± 1.0 vs. 1.0 ± 0.52 mg/dL; p < 0.001), higher INR (1.7 ± 0.39 vs. 1.4 ± 0.35; p < 0.001), higher total bilirubin (9.0 ± 9.73 vs. 3.1 ± 4.10 mg/dL; p < 0.001), lower hemoglobin (9.6 ± 1.76 vs. 10.3 ± 1.51 g/dL; p = 0.002), history of chronic kidney disease (28% vs. 13%; p = 0.008), history of spontaneous bacterial peritonitis (11% vs. 4%; p = 0.05), and were more likely to have TIPS placed emergently (41% vs. 18%; p < 0.001).

Among patients with refractory ascites, those who died had higher total bilirubin (5.4 ± 7.47 vs. 2.1 ± 2.05; p = 0.007), higher creatinine (1.9 ± 1.01 vs. 1.4 ± 0.89 mg/dL; p = 0.006), older age (61 ± 10.5 vs. 57 ± 9.9 years; p = 0.01), and use of an uncovered stent (47% vs. 29%; p = 0.04).

### Comparison of C statistics of prognostic scores

The AUROCs/C statistics for prediction of 90-day mortality for each prognostic score are presented in **Table 4**. Among all 413 patients, MELD score best predicted mortality (c = 0.779). Its performance was similar to MELD-Na score (c = 0.767; p = 0.42). MELD score outperformed CLIF-C ACLF score (c = 0.695; p = 0.03), CP score (c = 0.673; p < 0.001), PALBI score (c = 0.712; p = 0.02), and Emory score (c = 0.667; p < 0.001).

**Table 4:** AUROCs of 90-day mortality prediction models.

**Patient Subgroup**	**All Patients** (n = 413)	**Variceal Bleeding** (n = 248)	**Refractory Ascites** (n = 165)	**Emergent TIPS**^¶^ (n = 61)	**Elective TIPS** (n = 187)	**Uncovered Stent** (n = 156)	**Covered Stent** (n = 254)
**Prediction Scores**							
MELD score	0.779 [0.729, 0.829]	0.844 [0.793, 0.895]	0.673 [0.575, 0.772]	0.817 [0.708, 0.927]	0.836 [0.771, 0.901]	0.732 [0.651, 0.813]	0.795 [0.725, 0.864]
MELD-Na score	0.767 [0.718, 0.816]	0.824 [0.771, 0.877]	0.689 [0.596, 0.782]	0.787 [0.671, 0.903]	0.820 [0.753, 0.886]	0.712 [0.630, 0.793]	0.793 [0.727, 0.858]
CLIF-C ACLF score	0.695 [0.636, 0.754]	0.687 [0.613, 0.762]	0.707 [0.608, 0.807]	0.665 [0.522, 0.807]	0.705 [0.615, 0.796]	0.657 [0.567, 0.748]	0.728 [0.648, 0.808]
Child-Pugh score^§^	0.673 [0.617, 0.730]	0.713 [0.642, 0.783]	0.640 [0.550, 0.730]	0.602 [0.459, 0.746]	0.722 [0.631, 0.814]	0.583 [0.485, 0.680]	0.714 [0.641, 0.787]
Platelet-Albumin-Bilirubin score	0.712 [0.654, 0.771]	0.750 [0.681, 0.819]	0.660 [0.559, 0.760]	0.648 [0.503, 0.793]	0.781 [0.703, 0.858]	0.649 [0.553, 0.744]	0.744 [0.668, 0.820]
Emory Score^§^	0.667 [0.605, 0.731]	0.709 [0.627, 0.791]	0.623 [0.530, 0.717]	0.585 [0.448, 0.722]	0.640 [0.511, 0.757]	0.614 [0.513, 0.714]	0.689 [0.606, 0.772]
**Individual Variables**							
Age	0.559 [0.496, 0.622]	0.524 [0.444, 0.605]	0.622 [0.522, 0.721]	0.503 [0.353, 0.654]	0.566 [0.471, 0.661]	0.591 [0.494, 0.688]	0.549 [0.466, 0.633]
Hemoglobin	0.592 [0.531, 0.653]	0.621 [0.593, 0.702]	0.547 [0.454, 0.639]	0.601 [0.453, 0.748]	0.656 [0.558, 0.754]	0.544 [0.448, 0.640]	0.640 [0.562, 0.719]
Sodium	0.532 [0.464, 0.599]	0.579 [0.493, 0.664]	0.538 [0.433, 0.642]	0.568 [0.421, 0.716]	0.570 [0.457, 0.683]	0.504 [0.409, 0600]	0.549 [0.450, 0.647]
Creatinine	0.721 [0.663, 0.779]	0.773 [0.705, 0.841]	0.667 [0.560, 0.774]	0.781 [0.660, 0.901]	0.756 [0.666, 0.847]	0.734 [0.650, 0.818]	0.705 [0.620, 0.789]
International normalized ratio	0.674 [0.614, 0.734]	0.731 [0.660, 0.802]	0.590 [0.487, 0.623]	0.681 [0.544, 0.819]	0.711 [0.616, 0.805]	0.574 [0.479, 0.668]	0.746 [0.668, 0.824]
Total bilirubin	0.708 [0.649, 0.770]	0.746 [0.676, 0.816]	0.654 [0.554, 0.754]	0.661 [0.516, 0.805]	0.770 [0.687, 0.853]	0.659 [0.564, 0.755]	0.731 [0.653, 0.808]

Area under the receiver operating characteristic values are displayed with [95% confidence intervals]

Key: AUROC (area under the receiver operating characteristic), TIPS (transjugular intrahepatic portosystemic shunt), MELD (Model for End Stage Liver Disease), CLIF-C ACLF (Chronic Liver Failure Consortium Organ Failure Acute on Chronic Liver Failure Score

^**§**^ n = 389

^**¶**^ Emergent placement was only considered among patients receiving TIPS for variceal bleeding.

Among 248 patients with variceal bleeding, MELD score best predicted mortality (c = 0.844). Its performance was similar to MELD-Na score and it outperformed all other scores. Among 165 patients with refractory ascites, CLIF-C ACLF score best predicted mortality (c = 0.707), though its performance was statistically similar to all other scores. Among 61 patients who received emergent TIPS for bleeding, MELD score best predicted mortality (c = 0.817) and it outperformed all other scores. Among 187 patients who received elective TIPS for bleeding, MELD score best predicted mortality (c = 0.836). Its performance was similar to that of MELD-Na score and it outperformed all other scores. Among 156 patients where an uncovered stent was used, MELD score best predicted mortality (c = 0.732). Among 254 patients where a covered stent was used, MELD score best predicted mortality (c = 0.795).

### Added predictive value of sodium, hemoglobin, and age to the meld score

With MELD score as the reference, the added predictive value of three variables was assessed: hemoglobin and age (the strongest non-MELD predictors of mortality for bleeding and ascites subgroups, respectively), as well as sodium. Addition of sodium to the MELD score did not significantly improve the AUROC, category-free NRI, or IDI for all patients or in any patient strata, except for category-free NRI among patients receiving an emergent TIPS for variceal bleeding (0.55; p = 0.03). Addition of hemoglobin to the MELD score improved its predictive value for all patients (IDI 0.00584; p = 0.05), variceal bleeding (NRI 0.35; p = 0.02 and IDI 0.033; p = 0.01), elective TIPS (NRI 0.40; p = 0.02 and IDI 0.05432; p = 0.01), and with covered stents (NRI 0.32; p = 0.03). Addition of age to the MELD score improved its predictive value for all patients (NRI 0.25; p = 0.02 and IDI 0.013853; p = 0.05) and refractory ascites (NRI 0.43; p = 0.01 and IDI 0.05274; p = 0.001; see **Table 5**).

For patients with refractory ascites, the risk of 90-day mortality can be modeled for MELD score and age as:

0.118*[MELDscore]+0.063*[Age]–6.668

For patients with variceal bleeding, the risk of 90-day mortality can be modeled for MELD score and hemoglobin as:

0.181*[MELDscore]–0.320*[Hemoglobin]–0.753

**Table 5:** Added predictive value for 90-day survival when adding serum sodium, hemoglobin, and age to MELD score, stratified by: Indication for TIPS, emergent versus elective status, and uncovered versus covered stent type.

	MELD	MELD + Na		P value	MELD + Hgb	P value	MELD + Age		P value
**All Patients** (n = 413)									
AUROC	0.779	0.779	0.88		0.785	0.41	0.800	0.21	
Category-free NRI		-0.08	0.45		0.12	0.28	0.25	0.02	
IDI		0.00066	0.35		0.00584	0.05	0.013853	0.05	
**Variceal Bleeding** (n = 248)									
AUROC	0.844	0.847	0.39		0.853	0.51	0.854	0.11	
Category-free NRI		0.13	0.35		0.35	0.02	0.15	0.28	
IDI		0.00178	0.58		0.03300	0.01	0.00504	0.35	
**Refractory Ascites** (n = 165)									
AUROC	0.673	0.673	0.99		0.675	0.63	0.731	0.14	
Category-free NRI		0.21	0.23		0.03	0.87	0.43	0.01	
IDI		0.00152	0.76		0.00007	0.95	0.05274	0.001	
**Emergent TIPS** (n = 61)									
AUROC	0.817	0.822	0.82		0.819	0.90	0.824	0.63	
Category-free NRI		0.55	0.03		0.09	0.72	0.08	0.76	
IDI		0.01490	0.41		0.01240	0.40	0.00953	0.31	
**Elective TIPS** (n = 187)									
AUROC	0.836	0.836	0.57		0.850	0.56	0.852	0.18	
Category-free NRI		0.10	0.57		0.40	0.02	0.20	0.27	
IDI		-0.00004	0.95		0.05432	0.01	0.00787	0.35	
**Uncovered Stent** (n = 156)									
AUROC	0.732	0.735	0.32		0.734	0.85	0.761	0.23	
Category-free NRI		-0.08	0.65		0.11	0.51	0.21	0.21	
IDI		0.00060	0.60		0.00849	0.24	0.01994	0.14	
**Covered Stent** (n = 254)									
AUROC	0.795	0.794	0.63		0.811	0.12	0.811	0.16	
Category-free NRI		-0.11	0.47		0.32	0.03	0.23	0.13	
IDI		0.00086	0.44		0.01314	0.15	0.01394	0.12	

Key: MELD (Model for End-Stage Liver Disease), TIPS (transjugular intrahepatic portosystemic shunt), Na (sodium), Hgb (hemoglobin), AUROC (area under the receiver operating characteristic), NRI (net reclassification index), IDI (integrated discrimination increment)

### Other outcomes and sensitivity analyses

95/389 (24%) patients were listed for liver transplant (excluding those with unknown listing status). 53/95 (56%) of listed patients received a liver transplant, including 14 patients during the 90-day follow-up period. Prognostic scores performed similarly when predicting 90-day death or liver transplant compared to death alone (see **S2 Table**). There was no difference in 90-day transplant-free survival between patients with bleeding and ascites (p = 0.73; see **S1 Fig**). At one year, overall mortality was 113/413 (40%). Prognostic scores performed similarly when predicting mortality at one year compared to 90 days, though AUROC was lower across all scores (see **S2 Table**). Among those with available data, 150/380 (39%) experienced hepatic encephalopathy and 49/399 (12%) required a TIPS revision (for technical reasons or due to encephalopathy) within 90 days of TIPS placement.

In sensitivity analysis, 20 patients with hepatic hydrothorax were similar to those with ascites alone (n = 145), including in age, sex, race, ethnicity, serum sodium, creatinine, hemoglobin, and pre/post-TIPS portosystemic gradient. Patients with hepatic hydrothorax were less likely to have CKD, had lower platelet counts, higher total bilirubin, and higher MELD scores (19 ± 8.4 vs. 15 ± 7.2; p = 0.02; see **S3 Table).** Mortality at 90 days was similar between those with hydrothorax and those with refractory ascites (30% vs. 26%; p = 0.72). Exclusion of the 20 patients with hydrothorax did not meaningfully change the C statistics for 90-day mortality of the six prognostic scores.

## Discussion

In a large population of 413 patients with cirrhosis who received TIPS for variceal bleeding or refractory ascites, we provide a detailed analysis of six widely available prognostic models for 90-day mortality, including the recently adopted MELD-Na and CLIF-C ACLF scores. To our knowledge, this is the first time CLIF-C ACLF score has been assessed among TIPS recipients. We found that MELD score performed best in predicting post-TIPS mortality, and that the addition of sodium to the MELD score did not significantly improve its prognostic ability. However, in specific subgroups, the prognostic ability of the MELD score could still be improved by adding important clinical variables, such as “Hgb-MELD” in those with variceal bleeding and “Age-MELD” in those with refractory ascites.

Overall, all six prognostic scores significantly predicted 90-day mortality, as well as one year mortality and 90-day transplant-free survival in sensitivity analyses. This is not surprising, as there are many overlapping components to these scores. We presented three measures of model performance in our analysis: direct change in AUROC, NRI, and IDI. There is no universally accepted method for assessing added usefulness of new metrics to existing predictive models. While a change in AUROC is perhaps most concrete in describing model performance, NRI and IDI may be more sensitive to detect incremental improvement.[20] In most subgroups of our study, MELD score generally performed best, followed closely by MELD-Na score. In patients with refractory ascites, CLIF-C ACLF score had the best C statistic, though it was not statistically significantly better than MELD or MELD-Na scores. When comparing MELD and MELD-Na scores more directly, addition of sodium did not have added predictive value, and MELD-Na score did not perform better than MELD score alone in any stratum. There may be two reasons for this. First, unlike in overall survival in cirrhosis, where MELD-Na score demonstrates prognostic improvement compared to MELD score,[8] patients referred for TIPS may be uniquely selected such that the influence of serum sodium is not as apparent. Second, there may be specific demographic and geographic factors that lessen the impact of sodium. All hospitals in this study were in Organ Procurement and Transplant Network (OPTN) Region 1, which has a higher median MELD score at the time of liver transplantation than most other regions in the United States.[21–23] This may also affect timing or selection for TIPS referral in ways not easily captured from clinical data.

In their cornerstone study evaluating the predictive value of liver disease scores after TIPS placement, Gaba *et al*. examined prognostic scores in a similar group of 211 TIPS recipients out of a single center in OPTN Region 7.[24] In their thorough analysis, these authors came to similar conclusions: namely, that MELD and MELD-Na score performed best overall. There are several differences to highlight between our two studies. First, scores in their study had better performance overall compared to this one, including MELD score (c = 0.816 for Gaba *et al*. compared to c = 0.779 in our study), which highlights the variable performance of MELD based on geography and local referral patterns. Second, their models had better performance for uncovered stents, whereas performance was better for covered stents in our study. This observation is less well-explained, but may also be due to population differences. Finally, while they conclude that MELD and MELD-Na performed similarly, we extend their conclusions by comparing these two scores more directly, using NRI and IDI to assess sodium’s added predictive value to MELD. Given the lack of statistical difference between direct comparisons of MELD and MELD-Na models in Chi square analysis, as well as the failure to reject the null hypothesis of NRI and IDI testing, we conclude that addition of sodium does not improve the performance of MELD score among TIPS recipients. While it would be ideal to have a single study comparing centers in multiple transplant regions to help address these differences in populations, it is reassuring that our results corroborate those of Gaba *et al*, adding to the generalizability of both results.

The original derivation and clinical application of each prognostic score may help better contextualize their statistical performances. CP score was the oldest score analyzed, as it was developed from 38 patients taken for emergent surgical ligation of bleeding varices in 1973.[10] Despite its vintage, it has routinely performed well in populations receiving TIPS.[12, 25] Three scores were more recently designed specifically to predict post-TIPS mortality: MELD score,[7] Emory score,[12] and PALBI score.[11] Among these three, MELD clearly performed the best. CLIF-C ACLF score was validated to predict mortality in patients hospitalized with acute-on-chronic liver failure.[9] Perhaps expectedly, the CLIF-C ACLF did not outperform MELD, as those with acutely decompensated cirrhosis are often not considered for TIPS placement, except in cases of emergent bleeding.

One important observation was that model performance varied by patient subgroup. Small differences in model performance were present between covered and uncovered stent types, though overall conclusions remained the same. This likely reflects improvements in practice patterns and TIPS placement technique over time, as uncovered stents were largely phased out by the midpoint of the study. Results from those receiving covered stents can be interpreted as a sensitivity analysis restricted to a more recent patient cohort. In general, prognostic scores had higher C statistics in patients with variceal bleeding compared to those with refractory ascites, suggesting that there may be other factors contributing to the morbidity of refractory ascites that may be not captured with our current prognostic tools. The addition of age improved MELD score’s performance among those with ascites, and may be another clinical consideration when making treatment decisions in this population. There are many factors to weigh when considering TIPS candidacy, including the overall survival benefit seen in early TIPS placement for both variceal bleeding and refractory ascites.[2, 3] Overall, we would recommend clinicians continue to take a holistic and evidence-based approach when evaluating patients for TIPS placement, using scores like MELD as a guide, but not as the absolute arbiter of candidacy.

This paper should be viewed in the context of its limitations. This is a retrospective study, and as such, findings should be viewed as associations, rather than as causal relationships. All sites were located in OPTN Region 1, which colors the practice patterns of referring providers and may not reflect experiences in other transplant regions. This is a common limitation of the TIPS literature, as there is no detailed national clinical registry for TIPS outcomes (beyond the limited datasets available from payer networks, such as the National Inpatient Sample).[26] Our study is the largest of its kind, and we believe it provides an important reference point for other regions to assess and validate. Unavailable data limited the calculation of CP and Emory scores for 24 patients, but we believe this analysis was sufficiently large and comprehensive to convey its key messages. Cause of death was also not available, which may introduce bias in the performance of the scores.

Among six prognostic models, MELD score best predicted 90-day mortality. Addition of sodium to the MELD score did not improve its predictive value. Significant predictors in the subgroups of variceal bleeding (hemoglobin) and refractory ascites (age) did improve MELD’s predictive ability in their respective strata. An individualized risk stratification approach, including modifications like an “Age-MELD” or “Hgb-MELD” score for appropriate subgroups, may be best when considering candidates for TIPS placement. A nationwide registry of post-TIPS outcomes is needed to better generalize these findings.

## Supporting information

S1 Fig Kaplan Meier curves visualizing (Panel A) 90-day survival and (Panel B) 90-day transplant-free survival among patients receiving TIPS for refractory ascites (red) versus variceal bleeding (blue).
(TIFF)

S1 Table Components and formulas of prognostic scores. Key: MELD (Model for End Stage Liver Disease), CLIF-C ACLF (Chronic Liver Failure Consortium Organ Failure Acute on Chronic Liver Failure Score; INR (international normalized ratio), MAP (mean arterial pressure), ALT (Alanine aminotransferase), TIPS (transjugular intrahepatic portosystemic shunt). ^a^ Respiratory organ failure simplified due to available clinical data.
(DOCX)

S2 Table AUROCs of prediction models for all patients’ mortality at 90 days, death or liver transplant at 90 days, and mortality at one year. Area under the receiver operating characteristic values are displayed with [95% confidence intervals] Key: AUROC (area under the receiver operating characteristic), MELD (Model for End Stage Liver Disease), CLIF-C ACLF (Chronic Liver Failure Consortium Organ Failure Acute on Chronic Liver Failure Score ^**§**^ n = 389.
(DOCX)

S3 Table Comparison of demographics and clinical characteristics of TIPS recipients with refractory ascites and hepatic hydrothorax. Continuous variables are displayed as mean ± standard deviation. Key: TIPS (transjugular intrahepatic portosystemic shunt), MELD (Model for End Stage Liver Disease), CLIF-C ACLF (Chronic Liver Failure Consortium Organ Failure Acute on Chronic Liver Failure Score. ^a^ n = 161; ^b^ n = 164; ^c^ n = 154; ^d^ n = 159; ^e^ n = 156; ^f^ n = 163; ^g^ n = 162.
(DOCX)

## Abbreviations

TIPS: transjugular intrahepatic portosystemic shunt
MELD: Model for End Stage Liver Disease
CLIF-C ACLF: Chronic Liver Failure Consortium Acute on Chronic Liver Failure
CP: Child-Pugh
PALBI: Platelet-Albumin-Bilirubin
AUROC: area under the receiver operating characteristic
NRI: net reclassification index
IDI: integrated discrimination index
CI: confidence intervals
Hgb: hemoglobin
OPTN: Organ Procurement and Transplant Network
