# Supplementary figures and images for: Evaluation of model performance to predict survival after transjugular intrahepatic portosystemic shunt placement
Source: PLoS One. 2019 May 23;14(5):e0217442. doi: 10.1371/journal.pone.0217442 (PMC6533008; doi:10.1371/journal.pone.0217442)

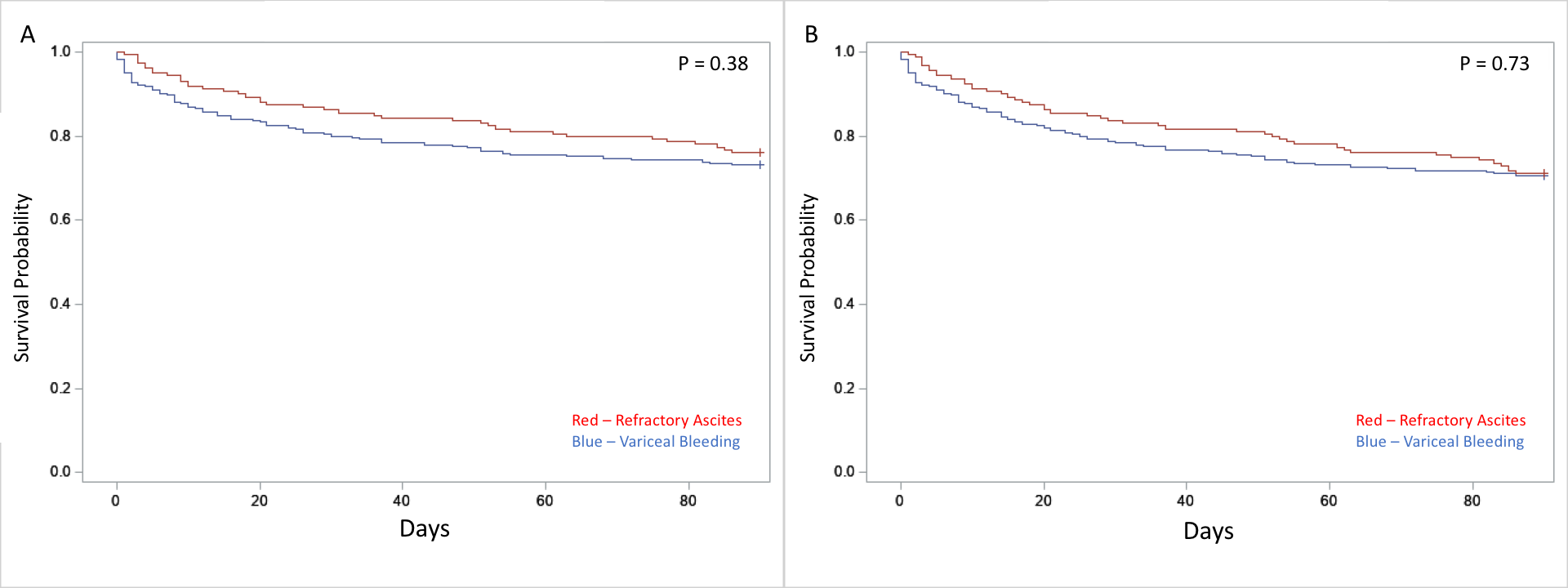

Supplement: S1 Fig — Kaplan Meier curves visualizing (Panel A) 90-day survival and (Panel B) 90-day transplant-free survival among patients receiving TIPS for refractory ascites (red) versus variceal bleeding (blue). (TIFF) [file pone.0217442.s001.tiff]
