# Supplementary material for: Evaluation of model performance to predict survival after transjugular intrahepatic portosystemic shunt placement
Source: PLoS One. 2019 May 23;14(5):e0217442. doi: 10.1371/journal.pone.0217442 (PMC6533008; doi:10.1371/journal.pone.0217442)
Supplement: S1 Table — Key: MELD (Model for End Stage Liver Disease), CLIF-C ACLF (Chronic Liver Failure Consortium Organ Failure Acute on Chronic Liver Failure Score; INR (international normalized ratio), MAP (mean arterial pressure), ALT (Alanine aminotransferase), TIPS (transjugular intrahepatic portosystemic shunt). a Respiratory organ failure simplified due to available clinical data. (DOCX) [file pone.0217442.s002.docx]

**S1 Table. Components and Formulas of Prognostic Scores**

| Model | Formula/Components |
| --- | --- |
| MELD | (0.957 * ln[serum creatinine] + 0.378 * ln[total bilirubin] + 1.120 * ln[INR] + 0.643) * 10  *If hemodialysis required, serum creatinine set to its upper limit (4.0)* |
| MELD-Na | MELD Score – [serum sodium] - 0.025 * MELD score * (140 – [serum sodium]) + 140  *Serum sodium outside 125-140 is set to nearest limit* |
| CLIF-C ACLF | 10 * (0.33 * [CLIF Organ Failure Score] + 0.04 * [age] + 0.63 * ln[white blood count] - 2)  *CLIF Organ Failure Score*  *1 point: bilirubin < 6 mg/dL, creatinine < 2.0 mg/dL, no encephalopathy, INR < 2.0, MAP > 70, normal respiratory function* ^a^  *2 points: bilirubin 6-12 mg/dL, creatinine 2.0-3.5 mg/dL, grade I-II encephalopathy, INR 2.0-2.5, MAP < 70*  *3 points: bilirubin > 12 mg/dL, creatinine > 3.5 mg/dL (or dialysis), grade III-IV encephalopathy, INR > 2.5, required vasopressors, required intubation for respiratory failure* ^a^ |
| Child-Pugh | 1 point: bilirubin < 2 mg/dL, albumin > 3.5 g/dL, INR < 1.7, no ascites, no encephalopathy  2 points: bilirubin 2-3 mg/dL, albumin 2.8-3.5 g/dL, INR 1.7-2.3, mild-moderate ascites, mild encephalopathy  3 points: bilirubin > 3 mg/dL, albumin < 2.8 g/dL, INR > 2.3, refractory ascites, severe encephalopathy |
| Platelet-Albumin-Bilirubin | 2.02 * log(total bilirubin) – 0.37 * (log[total bilirubin])^2^ – 0.04 * albumin –3.48 * log(platelets) + 1.01 * (log[platelets])^2^ |
| Emory | 2 points: emergent TIPS  1 point: ALT > 100 units/L, bilirubin > 3.0 mg/dL, pre-TIPS encephalopathy |

Key: MELD (Model for End Stage Liver Disease), CLIF-C ACLF (Chronic Liver Failure Consortium Organ Failure Acute on Chronic Liver Failure Score; INR (international normalized ratio), MAP (mean arterial pressure), ALT (Alanine aminotransferase**),** TIPS (transjugular intrahepatic portosystemic shunt)

^a^ Respiratory organ failure simplified due to available clinical data
