# Supplementary material for: Evaluation of model performance to predict survival after transjugular intrahepatic portosystemic shunt placement
Source: PLoS One. 2019 May 23;14(5):e0217442. doi: 10.1371/journal.pone.0217442 (PMC6533008; doi:10.1371/journal.pone.0217442)
Supplement: S2 Table — Area under the receiver operating characteristic values are displayed with [95% confidence intervals] Key: AUROC (area under the receiver operating characteristic), MELD (Model for End Stage Liver Disease), CLIF-C ACLF (Chronic Liver Failure Consortium Organ Failure Acute on Chronic Liver Failure Score § n = 389. (DOCX) [file pone.0217442.s003.docx]

**S2 Table. AUROCs of prediction models for all patients’ mortality at 90 days, death or liver transplant at 90 days, and mortality at one year**

| Prediction Scores | 90 Day Mortality | 90 Day Death or Liver Transplant | One Year Mortality |
| --- | --- | --- | --- |
| MELD score | 0.779 [0.729, 0.829] | 0.787 [0.740, 0.835] | 0.753 [0.706, 0.800] |
| MELD-Na score | 0.767 [0.718, 0.816] | 0.788 [0.742, 0.834] | 0.755 [0.708, 0.801] |
| CLIF-C ACLF score | 0.695 [0.636, 0.754] | 0.665 [0.606, 0.724] | 0.654 [0.600, 0.708] |
| Child-Pugh score^§^ | 0.673 [0.617, 0.730] | 0.707 [0.653, 0.760] | 0.646 [0.591, 0.700] |
| Platelet-Albumin-Bilirubin score | 0.712 [0.654, 0.771] | 0.726 [0.671, 0.781] | 0.663 [0.609, 0.716] |
| Emory Score^§^ | 0.667 [0.605, 0.731] | 0.688 [0.632, 0.743] | 0.592 [0.536, 0.648] |

Area under the receiver operating characteristic values are displayed with [95% confidence intervals]

Key: AUROC (area under the receiver operating characteristic), MELD (Model for End Stage Liver Disease), CLIF-C ACLF (Chronic Liver Failure Consortium Organ Failure Acute on Chronic Liver Failure Score

**^§^** n = 389
