# Supplementary material for: Evaluation of model performance to predict survival after transjugular intrahepatic portosystemic shunt placement
Source: PLoS One. 2019 May 23;14(5):e0217442. doi: 10.1371/journal.pone.0217442 (PMC6533008; doi:10.1371/journal.pone.0217442)
Supplement: S3 Table — Continuous variables are displayed as mean ± standard deviation. Key: TIPS (transjugular intrahepatic portosystemic shunt), MELD (Model for End Stage Liver Disease), CLIF-C ACLF (Chronic Liver Failure Consortium Organ Failure Acute on Chronic Liver Failure Score. a n = 161; b n = 164; c n = 154; d n = 159; e n = 156; f n = 163; g n = 162. (DOCX) [file pone.0217442.s004.docx]

**S3 Table. Comparison of demographics and clinical characteristics of TIPS recipients with refractory ascites and hepatic hydrothorax**

|  | Ascites Alone  (n = 145) | Hepatic Hydrothorax  (n = 20) | P value |
| --- | --- | --- | --- |
| Age (years) | 58 ± 10.4 | 56 ± 8.9 | 0.43 |
| Female sex (%) | 52 (36%) | 4 (20%) | 0.16 |
| White race (%) | 121 (83%) | 18 (90%) | 0.45 |
| Non-Hispanic ethnicity (%) | 137 (94%) | 19 (95%) | 0.92 |
| Diabetes mellitus (%) ^a^ | 52 (37%) | 4 (21%) | 0.18 |
| Chronic kidney disease (%) ^a^ | 42 (30%) | 1 (5%) | 0.02 |
| Etiology of cirrhosis (%) ^b^ | -- | -- | 0.05 |
| Prior complications of liver disease (%) |  |  |  |
| Pre-TIPS encephalopathy ^c^ | 56 (42%) | 7 (35%) | 0.56 |
| Gastrointestinal bleeding ^d^ | 57 (40%) | 6 (33%) | 0.56 |
| Spontaneous bacterial peritonitis ^e^ | 32 (23%) | 2 (11%) | 0.20 |
| Laboratory values |  |  |  |
| Sodium (mEq/L) | 134 ± 5.3 | 133 ± 6.8 | 0.64 |
| Creatinine (mg/dL) | 1.6 ± 0.90 | 1.6 ± 1.24 | 0.81 |
| White blood count (K/uL) | 6.8 ± 3.89 | 7.4 ± 2.81 | 0.47 |
| Hemoglobin (g/dL) | 10.1 ± 1.58 | 10.5 ± 1.84 | 0.29 |
| Platelets (K/uL) | 123 ± 78.9 | 74 ± 30.6 | <0.001 |
| Albumin (g/dL) | 2.9 ± 0.56 | 2.7 ± 0.62 | 0.24 |
| International normalized ratio (INR) | 1.5 ± 0.40 | 1.7 ± 0.37 | 0.05 |
| Total bilirubin (mg/dL) | 2.7 ± 4.31 | 5.1 ± 4.96 | 0.02 |
| Aspartate aminotransferase (U/L) | 99 ± 163.5 | 84 ± 48.1 | 0.40 |
| Alanine aminotransferase (U/L) | 57 ± 95.1 | 46 ± 37.7 | 0.37 |
| Alkaline phosphatase (U/L) | 145 ± 99.9 | 152 ± 133.7 | 0.78 |
| TIPS procedural characteristics |  |  |  |
| Pre-TIPS portosystemic gradient (mm Hg) ^c^ | 17 ± 5.2 | 17 ± 5.0 | 0.95 |
| Post-TIPS portosystemic gradient (mm Hg) ^f^ | 7 ± 3.0 | 7 ± 2.9 | 0.27 |
| Reduction in portosystemic gradient (mm Hg) ^c^ | 11 ± 4.7 | 10 ± 4.4 | 0.47 |
| Covered stent (%) ^f^ | 95 (66%) | 13 (65%) | 0.90 |
| Diameter of final TIPS dilation (mm) ^g^ | 9.9 ± 0.97 | 10.4 ± 1.42 | 0.09 |
| Liver Disease Prediction Models |  |  |  |
| MELD score | 15 ± 7.2 | 19 ± 8.4 | 0.02 |
| MELD-Na score | 19 ± 6.5 | 23 ± 7.5 | 0.01 |
| CLIF-C ACLF score | 40 ± 6.5 | 42 ± 7.3 | 0.24 |
| Child-Pugh score ^c^ | 10 ± 1.8 | 11 ± 1.6 | 0.02 |
| Platelet-Albumin-Bilirubin score | -2.6 ± 0.70 | -2.1 ± 0.61 | 0.002 |
| Emory Score ^c^ | 0.8 ± 0.78 | 1.0 ± 0.86 | 0.25 |

Continuous variables are displayed as mean ± standard deviation.

Key: TIPS (transjugular intrahepatic portosystemic shunt), MELD (Model for End Stage Liver Disease), CLIF-C ACLF (Chronic Liver Failure Consortium Organ Failure Acute on Chronic Liver Failure Score

^a^ n = 161; ^b^ n = 164; ^c^ n = 154; ^d^ n = 159; ^e^ n = 156; ^f^ n = 163; ^g^ n = 162
